# P38/MAPK contributes to endothelial barrier dysfunction via MAP4 phosphorylation-dependent microtubule disassembly in inflammation-induced acute lung injury

**DOI:** 10.1038/srep08895

**Published:** 2015-03-09

**Authors:** Lingfei Li, Jiongyu Hu, Ting He, Qiong Zhang, Xu Yang, Xiaodong Lan, Dongxia Zhang, Hao Mei, Bing Chen, Yuesheng Huang

**Affiliations:** 1 Institute of Burn Research, State Key Laboratory of Trauma, Burns and Combined Injury, Southwest Hospital, Third Military Medical University, Chongqing, China; 2 Endocrinology Department, Southwest Hospital, Third Military Medical University, Chongqing, China; 3 Institute of Respiratory Diseases, Xinqiao Hospital, Third Military Medical University, Chongqing, China; 4 Department of Biostatistics in the School of Public Health, Yale University

## Abstract

Excessive activation of inflammation and the accompanying lung vascular endothelial barrier disruption are primary pathogenic features of acute lung injury (ALI). Microtubule-associated protein 4 (MAP4), a tubulin assembly-promoting protein, is important for maintaining the microtubule (MT) cytoskeleton and cell-cell junctional structures. However, both the involvement and exact mechanism of MAP4 in the development of endothelial barrier disruption in ALI remains unknown. In this study, lipopolysaccharide (LPS) and tumour necrosis factor-α (TNF-α) were applied to human pulmonary microvascular endothelial cells (HPMECs) to mimic the endothelial damage during inflammation *in vitro*. We demonstrated that the MAP4 (Ser696 and Ser787) phosphorylation increased concomitantly with the p38/MAPK pathway activation by the LPS and TNF-α stimulation of HPMECs, which induced MT disassembly followed by hyperpermeability. Moreover, the application of taxol, the overexpression of a MAP4 (Ala) mutant, or the application of the p38/MAPK inhibitor SB203580 inhibited the MT disruption and the intracellular junction dysfunction. In contrast, MKK6 (Glu), which constitutively activated p38/MAPK, resulted in microtubule depolymerisation and, subsequently, hyperpermeability. Our findings reveal a novel role of MAP4 in endothelial barrier dysfunction.

Acute lung injury (ALI) is characterised by increased lung vascular permeability, along with a reduced alveolar liquid clearance capacity; these changes typically develop in tandem and lead to progressive deterioration of lung function^1^. Severe inflammation leads to excessive neutrophil activation and the production of many inflammatory mediators, such as lipopolysaccharide (LPS) and tumour necrosis factor-α (TNF-α), which are causative agents implicated in the pathogenesis of ALI^2^,^3^,^4^. Given that all blood vessels are lined with endothelial cells, the vascular leakage and pulmonary oedema associated with inflammation suggest endothelial dysfunction^5^. Thus, elucidating the mechanisms that underlie the contribution of inflammatory mediators to lung endothelial barrier dysfunction may provide a novel clinical therapeutic target against ALI.

Microtubules (MTs), a key component of the cytoskeleton, are responsible for regulating many processes, including cell migration and mitosis, as well as for maintaining cell shape and organelle transport^6^,^7^,^8^. The altered dynamics of MT assembly/disassembly and spatial rearrangement are essential for increased endothelial permeability^6^,^9^,^10^. However, the effect and mechanism of the inflammatory mediator-induced instability of MT dynamics and the MT-dependent regulation of the inflammatory response of endothelial cells have not been determined.

Microtubule-associated protein 4 (MAP4) is primarily recognised as a cytosolic MT-binding protein that is ubiquitously expressed in non-neural cells and has an important role in microtubule dynamics, as indicated in previous studies by ourselves and other researchers^11^,^12^,^13^,^14^,^15^. Once it is phosphorylated, MAP4 dissociates from tubulin, resulting in MT instability^12^,^13^. We previously reported that p38/MAPK activation could lead to MAP4 phosphorylation with MT disassembly in cardiomyocytes during hypoxia^11^; furthermore, p38/MAPK is an essential signal required for the cytoskeleton rearrangement that precedes endothelial barrier disruption^16^,^17^. Therefore, it is conceivable that a potential regulatory balance between MAP4 phosphorylation and dephosphorylation controlled by p38/MAPK may exist to maintain MT organisation and endothelial barrier function under inflammatory conditions.

In this study, we investigated the role of MAP4-MT binding and the role of p38/MAPK signalling in endothelial cells in mediating lung vascular leakage and ALI. We found that LPS or TNF-α led to endothelial hyperpermeability due to MT depolymerisation in HPMECs by activating p38/MAPK and inducing MAP4 phosphorylation. Thus, MAP4 may play a major role in the maintenance of vascular integrity and may provide a new potential therapeutic strategy for decreasing hyperpermeability in ALI.

## Results

### Inflammation-induced endothelial barrier dysfunction and MT disassembly

**Figure 1:**
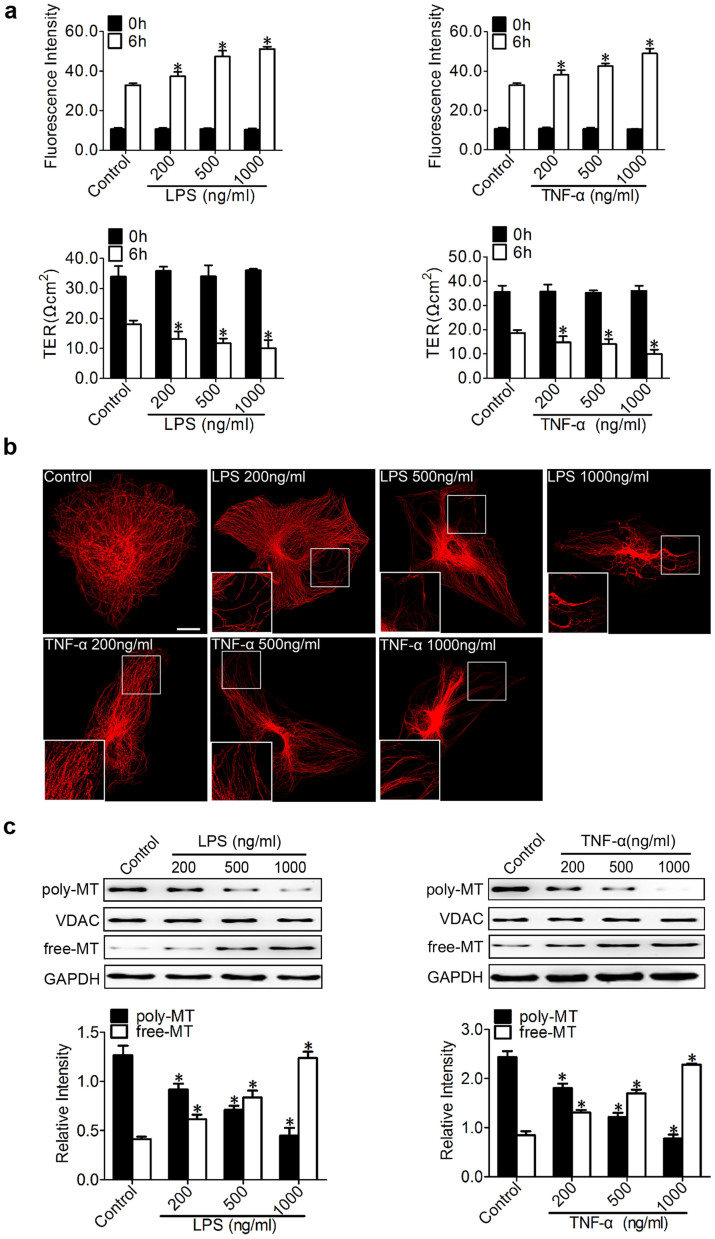
Inflammation-induced endothelial barrier dysfunction and MT disassembly. (a) The permeability of endothelial cells was assessed by measuring the influx of FITC-conjugated dextran and the transendothelial electrical resistance (TER) across the cells at 0 and 6 hr after treatment with 200, 500, and 1000 ng/ml LPS or TNF-α. The data are represented as the mean ± SEM (n = 3). *P < 0.05 vs. control group. (b) Representative confocal immunofluorescence images showing the microtubules (MTs) organisation of the control cells and the cells after treatment with 200, 500, and 1000 ng/ml of LPS or TNF-α. Bar, 10 μm. The inserts show high-magnification images of the peripheral MT network. (c) Western blot analysis was performed to detect poly/free tubulin after 6 hr of treatment with 200, 500, and 1000 ng/ml LPS or TNF-α. VDAC and GAPDH were used as the marker proteins for polymerised tubulin and free tubulin, respectively. The data are represented as the mean ± SEM (n = 3). *P < 0.05 vs. the control group.

We chose to study endothelial barrier function using HPMECs due to the critical importance of pulmonary vascular barrier function, and LPS and TNF-α were applied to HPMECs to mimic inflammatory pulmonary conditions *in vitro*. The permeability of the endothelial monolayer was tested by measuring the influx of FITC-conjugated dextran and the transendothelial electrical resistance (TER) across cells (Fig. 1a). LPS and TNF-α were found to induce endothelial barrier dysfunction, as represented by increased dextran leakage and decreased TER, in a dose-dependent manner (Fig. 1a).

In the morphological studies, the HPMECs subjected to LPS or TNF-α showed clear signs of MT disruption and modification (Fig. 1b). In quiescent endothelial cells, the MTs organised into a faint, uniformly distributed lattice network, whereas the LPS (200 ng/ml) challenge caused a less regular organisation and some breakages that changed the MT appearance. The disruption continued with the increase in LPS (500 ng/ml), and there was pronounced MT disassembly along the edges of the cell and some shrinkage in the vicinity of the nuclei compared with normal cells. With further LPS treatment (1000 ng/ml), only a thin and disordered residual MT network with some MT fragments remained (Fig. 1b, upper panel). The TNF-α treatment led to a similar, less regular organisation and MT breakage, as well as MT disassembly and reduced MT density; this change was as significant as that observed in association with the LPS treatment (Fig. 1b, lower panel). The insets in the micrographs of Figure 1 show high-magnification images of the broken microtubules after stimulation by LPS or TNF-α.

To further validate the effect of inflammatory stimulation on MTs, the tubulin fractions were isolated and analysed using Western blot analysis (Fig. 1c) to elucidate the changes in tubulin^11^. Our results consistently confirmed a concentration-dependent decrease in polymerised tubulin with a parallel increase in free tubulin after treatment with LPS and TNF-α (Fig. 1c). VDAC and GAPDH were used as the internal controls for polymerised tubulin and free tubulin, respectively.

### Involvement of MT disassembly in inflammation-induced endothelial permeability

**Figure 2:**
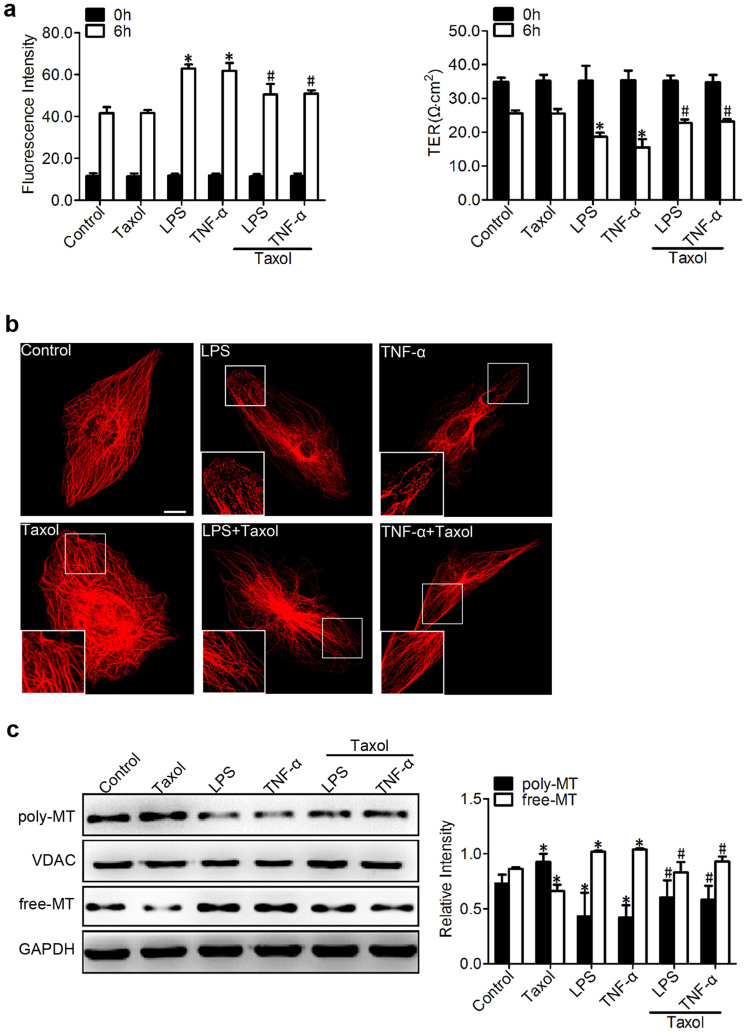
Involvement of MT disassembly in inflammation-induced endothelial permeability. (a) The permeability of endothelial cells was assessed by measuring the influx of FITC-conjugated dextran and the TER across the cells with and without taxol (1 μM, pretreated for 1 hr). The graph shows the mean ± SEM (n = 3). *P < 0.05 vs. the control group; ^#^P < 0.05 vs. the LPS or TNF-α group. (b) Confocal immunofluorescence images of MTs treated with LPS or TNF-α (500 ng/ml for 6 hr) with and without the addition of taxol. Bar, 10 μm. The inserts show high-magnification images of the peripheral MT network. (c) Western blotting was used to detect poly/free tubulin after treatment with LPS or TNF-α (500 ng/ml for 6 hr) with and without taxol. VDAC and GAPDH were used as the internal controls for polymerised tubulin and free tubulin, respectively. The graph shows the mean ± SEM (n = 3). *P < 0.05 vs. the control group; ^#^P < 0.05 vs. the taxol, LPS, or TNF-α group.

From the experiments above, LPS and TNF-α were found to trigger vascular barrier dysfunction accompanied by MT disruption (Fig. 1a, b and c). However, the roles of MTs in endothelial barrier regulation are much less defined. To determine whether the observed endothelial barrier dysfunction resulted from MT disruption, taxol was applied to stabilise the MT polymer and protect it from disassembly^18^. Our results revealed that taxol (1 μM) pretreatment significantly attenuated the LPS (500 ng/ml)- or TNF-α (500 ng/ml)-induced endothelial cell hyperpermeability (Fig. 2a), as indicated by the dextran leakage (1.25-fold reduction in LPS and 1.22-fold reduction in TNF-α) and the TER (1.22-fold increase in LPS and 1.49-fold increase in TNF-α) across monolayer cells, whereas taxol alone did not cause a significant change in dextran leakage and TER (Fig. 2a).

We then evaluated the role of a MT stabiliser in mediating the inflammation-induced MT disruption (Fig. 2b). These studies were performed using tubulin antibody. The taxol pretreatment led to preservation of the MT network and an increase in the density of the MT segments near the plasma cell membrane, in contrast to the expected LPS- and TNF-α-induced MT disassembly (Fig. 2b).

Concomitant with the decreased permeability and refined microtubule structure (Fig. 2a and 2b), the LPS- or TNF-α-induced change in tubulin fraction composition was alleviated by taxol as well (Fig. 2c). Western blot and quantitative analyses showed an increase in polymerised tubulin with a parallel decrease in free tubulin in response to LPS and TNF-α when pretreated with taxol; this finding indicated that taxol exerted a protective effect against LPS or TNF-α treatment (Fig. 2c).

### LPS- and TNF-α-induced MAP4 phosphorylation and its effect on endothelial barrier function and microtubules

**Figure 3:**
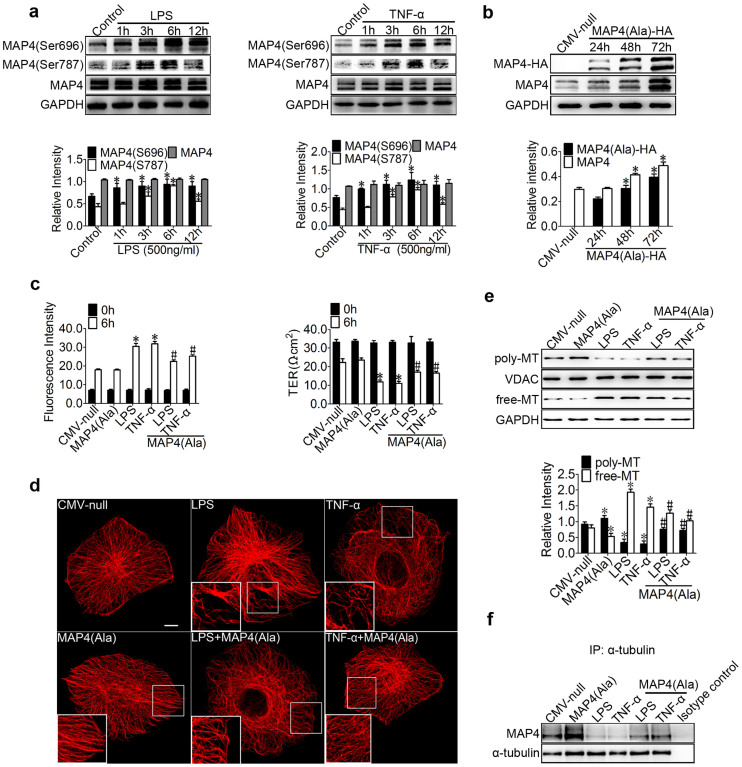
LPS- and TNF-α-induced MAP4 phosphorylation and its effect on endothelial barrier function and MTs. (a) HPMECs were treated with LPS or TNF-α (500 ng/ml) for 1, 3, 6, and 12 hr and examined using Western blotting. The graph shows the mean ± SEM (n = 3). *P < 0.05 vs. the control. (b) Confirmation of MAP4 (Ala) transfection at comparable levels in HPMECs. (c) The permeability of endothelial cells was assessed by measuring the influx of FITC-conjugated dextran and the TER. The graph shows the mean ± SEM (n = 3). *P < 0.05 vs. CMV-null; ^#^P < 0.05 vs. the LPS or TNF-α group. (d) For the immunofluorescence confocal micrographs, all the cells were transfected with CMV-null or MAP4 (Ala) and then treated or untreated with LPS or TNF-α for 6 hr. Bar, 10 μm. The inserts show high-magnification images of the peripheral MT network. (e) Cells were treated with LPS or TNF-α after being transfected with CMV-null or MAP4 (Ala). The Western blot shows polymerised and free tubulin; VDAC and GAPDH were used as the marker proteins. The graph shows the mean ± SEM (n = 3). *P < 0.05 vs. CMV-null; ^#^P < 0.05 vs. the MAP4 (Ala), LPS, or TNF-α group. (f) Determination of MAP4 binding to tubulin in cells with or without CMV-null or MAP4 (Ala) overexpression under LPS or TNF-α treatment by IP; Isotype control is as the negative control (n = 3).

We and others have determined that the amino acid residues S696, S768, and S787 in MAP4 are the critical sites responsible for the binding of MAP4 to tubulin; the phosphorylation of these sites leads to the dissociation of MAP4 from tubulin^12^,^13^,^19^,^20^. To determine whether the above observed MT disassembly (Fig. 1) was caused by MAP4 phosphorylation, the MAP4 phosphorylation was investigated using Western blots of HPMECs with or without LPS or TNF-α treatment (Fig. 3a). MAP4 was found to show weak basal levels of phosphorylation in cultured HPMECs and that LPS (500 ng/ml) or TNF-α (500 ng/ml) stimulation induced robust phosphorylation at S696 and S787 (Fig. 3a) in a time-dependent manner but not at S768 (Supplementary Fig. S1a); the total level of MAP4 remained unchanged before and after LPS or TNF-α treatment (Fig. 3a).

We next considered the possibility that MAP4 phosphorylation was important in mediating endothelial barrier dysfunction and MT disassembly in ALI after direct LPS or TNF-α exposure. We first constructed a MAP4 (Ala) mutant, which mimicked the non-phosphorylated form, by changing S696, S768, and S787 to alanine. HA-tagged MAP4 (Ala) or CMV-null was overexpressed at comparable levels in HPMECs and examined by Western blot analysis (Fig. 3b). The results demonstrated that the amount of escaping FITC-dextran was dramatically decreased (1.36- and 1.26-fold decrease compared with the value for LPS and TNF-α, respectively), and the TER across the endothelial cells was increased (1.44- and 1.49-fold increase compared with the value for LPS and TNF-α, respectively) in MAP4 (Ala) transfectants compared with that across cells transfected with CMV-null after LPS (500 ng/ml) or TNF-α (500 ng/ml) stimulation. In contrast, the MAP4 (Ala) transfectants alone did not alter the dextran leakage and TER (Fig. 3c).

To study whether the phosphorylation status of MAP4 influences MT structure, a MAP4 (Ala) mutant was transfected into cultured HPMECs (Fig. 3d). The effect of MAP4 (Ala) on MT structure under normal or inflammatory conditions was tested using immunofluorescence staining. MAP4 (Ala) was found to promote MT assembly under normal conditions and could protect against LPS- or TNF-α-induced MT depolymerisation (Fig. 3d). Similarly, MAP4 (Ala) also led to an increase in the amount of polymerised tubulin while decreasing the amount of free tubulin (Fig. 3e), as well as prevented the dissociation of MAP4 with tubulin (Fig. 3f) under LPS and TNF-α stimulation, and promoted the combination of MAP4 with tubulin compared with CMV-null (Fig. 3f), as assessed by Western blot analysis of the poly/free tubulin fractions (Fig. 3e) and immunoprecipitation (Fig. 3f), respectively. Collectively, these data confirmed that the phosphorylation of MAP4 at S696 and S787 promotes MT depolymerisation and the subsequent endothelial barrier dysfunction under inflammatory conditions in HPMECs.

### P38/MAPK activation mediates MAP4 phosphorylation in inflammation-induced acute lung injury

**Figure 4:**
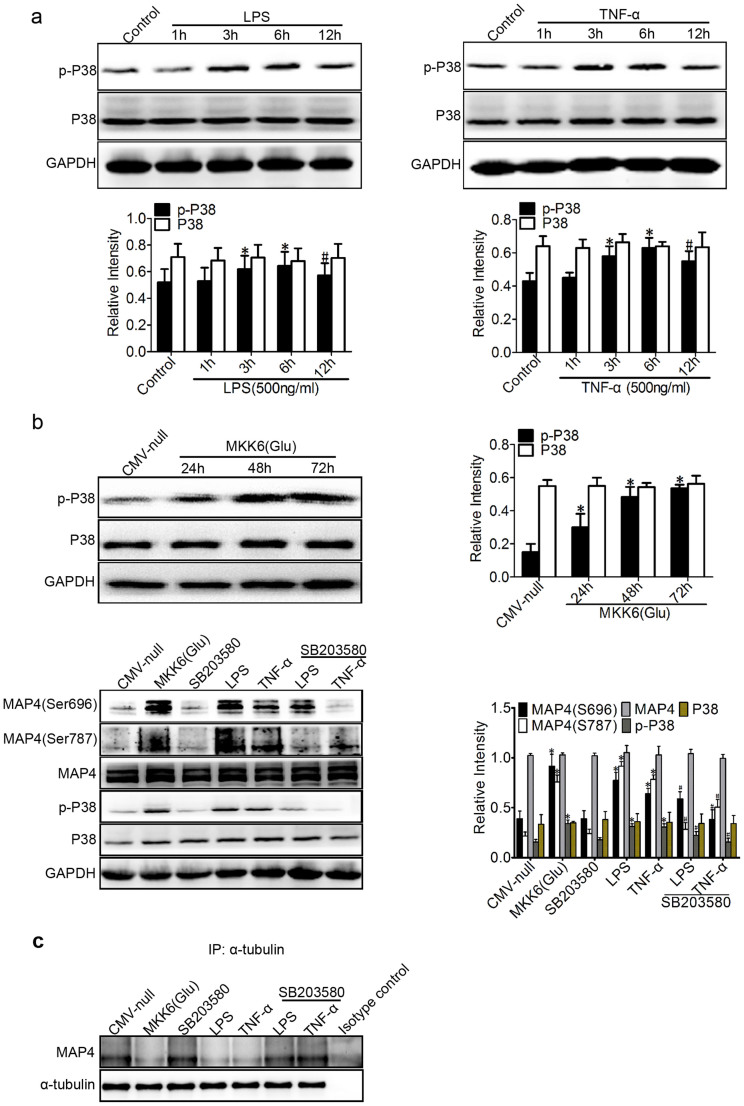
P38/MAPK activation mediates MAP4 phosphorylation in inflammation-induced ALI. (a) Western blotting was used to detect phospho-p38 (p-P38) and P38 following treatment with LPS or TNF-α (500 ng/ml for 1, 3, 6, and 12 hr). *P < 0.05 vs. the control group; ^#^P < 0.05 vs. the LPS/TNF-α (6 h) group. (b) Confirmation of MKK6 (Glu) transfection at comparable levels in HPMECs. Cells were transfected with CMV-null or MKK6 (Glu) and pretreated with SB203580 (5 μM) before the LPS or TNF-α treatment. The Western blot shows the phosphorylation of P38 and MAP4 at S696 and S787 and the total levels of MAP4 and P38. The graph shows the mean ± SEM (n = 3). *P < 0.05 vs. the CMV-null group; ^#^P < 0.05 vs. the LPS or TNF-α group. (c) Determination of MAP4 binding to tubulin in cells with or without MKK6 (Glu) overexpression or SB203580 pretreatment under LPS or TNF-α treatment by IP; Isotype control is as the negative control (n = 3).

P38/MAPK was identified as the upstream kinase of MAP4 phosphorylation in hypoxic cardiomyocytes in our previous study^11^. To elucidate whether the p38/MAPK signalling event is involved in inflammation-induced MAP4 phosphorylation, MT disassembly, and endothelial barrier dysfunction in HPMECs, we first investigated the activation of p38/MAPK under inflammatory stimulation (Fig. 4a). Activated p38/MAPK was assessed using Western blot analysis with a phosphor-specific p38/MAPK antibody (Thr180/Tyr182). The activation of p38/MAPK was significantly elevated from 3 to 12 hr after LPS (500 ng/ml) or TNF-α (500 ng/ml) treatment and peaked at 6 hr, with 1.25- and 1.49-fold increases, respectively (Fig. 4a).

We next tested whether the activation of p38/MAPK signalling was essential for MAP4 phosphorylation. MKK6 (Glu), a constitutively activated p38 kinase activator, was overexpressed in HPMECs to activate the p38/MAPK signalling pathway (Fig. 4b). Conversely, the p38/MAPK inhibitor SB203580 (5 μM) was applied to inhibit the p38/MAPK activation in HPMECs under the inflammatory challenge, and MAP4 phosphorylation (S696 and S787) was then measured (Fig. 4b). Western blot analysis and quantification confirmed that the MAP4 phosphorylation (S696 and S787) in the MKK6 (Glu) group increased (2.35- and 4.51-fold, respectively) significantly, and SB203580 (5 μM) decreased the MAP4 phosphorylation induced by LPS (1.21- and 2.23-fold reduction at S696 and S787, respectively) or TNF-α (1.67- and 1.55-fold reduction at S696 and S787, respectively) (Fig. 4b). Additionally, MKK6 (Glu) was also confirmed to show the dissociation of MAP4 with tubulin (Fig. 4c). In contrast, the p38/MAPK inhibitor was found to protect against such dissociation under LPS or TNF-α stimulation, as assessed by immunoprecipitation (Fig. 4c). These results indicate that p38/MAPK acts as the upstream kinase that promotes MAP4 phosphorylation in inflammation-induced ALI.

### Role of p38/MAPK activation in LPS- and TNF-α-induced endothelial barrier dysfunction and MT disassembly

Our previous work demonstrated that MAP4 phosphorylation induces MT disassembly and endothelial barrier dysfunction after LPS or TNF-α stimulation, and we identified p38/MAPK as the upstream kinase of MAP4 phosphorylation. However, whether the p38/MAPK is the pivotal kinase that mediates MT disassembly and endothelial barrier dysfunction under inflammatory conditions must still be determined.

**Figure 5:**
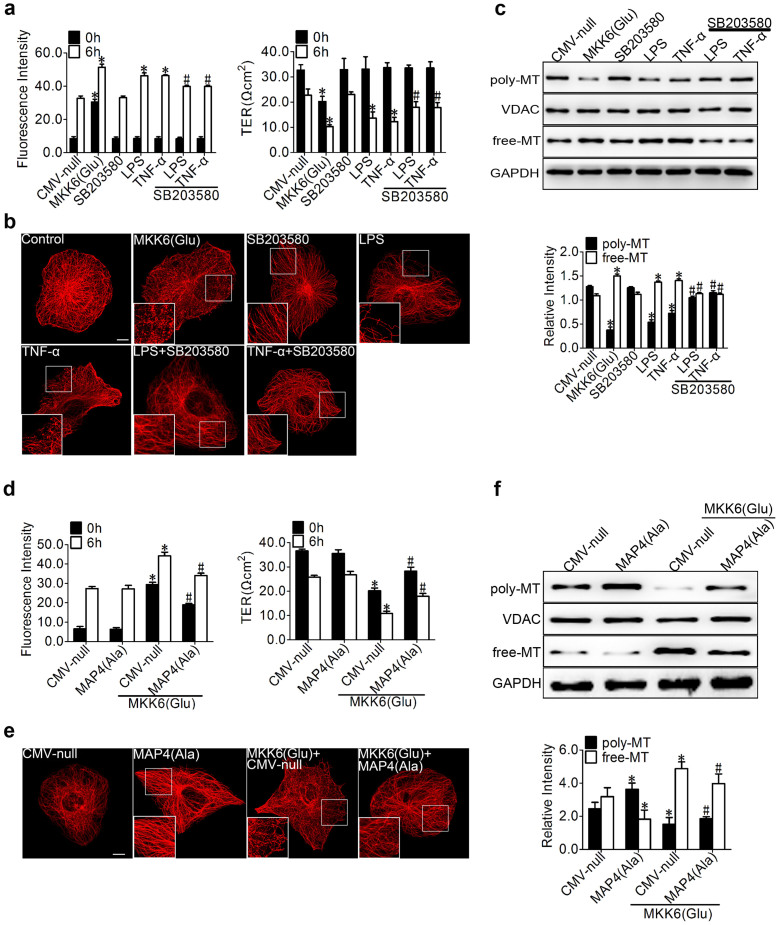
Role of p38/MAPK activation in LPS- and TNF-α-induced endothelial barrier dysfunction and MT disassembly. (a) Cells were pretreated with SB203580 (5 μM) for 1 hr, and CMV-null or MKK6 (Glu) was transfected into HPMECs for 72 hr before the LPS or TNF-α (500 ng/ml) treatment. The permeability of endothelial cells was assessed by measuring the influx of FITC-conjugated dextran and the TER across the cells. The data are represented as the mean ± SEM (n = 3). *P < 0.05 vs. CMV-null; ^#^P < 0.05 vs. the LPS or TNF-α group. (b) For the immunofluorescence confocal micrographs, HPMECs were transfected with CMV-null or MKK6 (Glu) and pretreated with or without SB203580 during an LPS or TNF-α challenge. (c) The Western blot shows poly/free tubulin with and without SB203580 pretreatment before an LPS or TNF-α challenge; MKK6 (Glu) was transfected into HPMECs for 72 hr before treatment. The data are represented as the mean ± SEM (n = 3). *P < 0.05 vs. CMV-null; ^#^P < 0.05 vs. the LPS or TNF-α group. (d) The HPMEC monolayer permeability was assessed by measuring the influx of FITC-conjugated dextran and the TER after the cells were transfected or cotransfected with CMV-null, MAP4 (Ala), or MKK6 (Glu). The data are represented as the mean ± SEM (n = 3). *P < 0.05 vs. the CMV-null and MAP4 (Ala) groups; ^#^P < 0.05 vs. the CMV-null + MKK6 (Glu) group. (e) Cells were studied using immunofluorescence staining with the anti-α-tubulin antibody. The boxed areas are shown at higher magnification in the inserts to provide a detailed illustration of the MTs. Bar, 10 μm. (f) Cells were transfected or cotransfected with CMV-null, MAP4 (Ala), or MKK6 (Glu). The poly/free tubulin fractions were determined by Western blot analysis; VDAC and GAPDH were used as the internal controls. The data are represented as the mean ± SEM (n = 3). *P < 0.05 vs. the CMV-null and MAP4 (Ala) groups; ^#^P < 0.05 vs. the CMV-null + MKK6 (Glu) group.

We then observed the effect of p38/MAPK activation and inhibition on endothelial barrier function and MT dynamics in HPMECs. SB203580 (5 uM) was found largely to abolish the LPS- or TNF-α-induced endothelial hyperpermeability, which resulted in 1.16- and 1.20-fold reduction in dextran leakage, and 1.31- and 1.46-fold increase in TER compared with those for LPS and TNF-α, respectively, whereas the transduction with MKK6 (Glu) under normal conditions resulted in hyperpermeability (3.59-fold increase in dextran leakage and 2.07-fold reduction in TER compared with CMV-null values) (Fig. 5a), suggesting the involvement of p38/MAPK in LPS- or TNF-α-induced endothelial barrier dysfunction. Concomitant with the prevention of hyperpermeability, SB203580 preserved the MT network in the LPS- or TNF-α-challenged cells (Fig. 5b), whereas the MKK6 (Glu) transfection resulted in complete destruction of the MT structure from the cell membrane to the nearby nuclei (Fig. 5b). Additionally, the quantification of the tubulin fractions (Fig. 5c) further confirmed that the transduction with MKK6 (Glu) led to significant MT depolymerisation, as represented by decreased polymerised tubulin and increased free tubulin. In contrast, the control cells expressing CMV-null showed no changes in their MT dynamics (Fig. 5c). Similarly, the SB203580 treatment significantly increased the amount of polymeric tubulin and decreased the amount of free tubulin under inflammatory conditions (Fig. 5c).

We next evaluated the critical role of MAP4 phosphorylation in mediating the p38/MAPK regulation of MT dynamics and endothelial barrier function (Fig. 5d, e and f). In this investigation, HPMECs were transiently cotransfected with MAP4 (Ala) and MKK6 (Glu), and the CMV-null was cotransfected with MKK6 (Glu) as the negative control of MAP4 (Ala). MAP4 (Ala) overexpression was found to prevent the p38/MAPK activation of endothelial barrier dysfunction (1.30-fold reduction in dextran leakage and 1.66-fold increase in TER compared with those for MKK6 (Glu) + CMV-null) (Fig. 5d). The HPMECs overexpressing MAP4 (Ala) were significantly more resistant than the CMV-null control to MT disassembly in response to MKK6 (Glu) transfection, as assessed by MT immunofluorescence staining and tubulin fraction analysis (Fig. 5e and 5f); this finding is consistent with our concept of a crucial role for MAP4 phosphorylation in p38/MAPK-mediated MT depolymerisation and endothelial barrier dysfunction.

## Discussion

In this study, we identified a novel role of MAP4 phosphorylation in endothelial cells in mediating the LPS- or TNF-α-induced increase in lung vascular permeability. We showed that LPS- or TNF-α induced MAP4 phosphorylation through activation of the p38/MAPK pathway in HPMECs, which led to MT disassembly and a parallel increase in vascular permeability, as indicated by the markedly increased dextran leakage and the decreased TER across the monolayer cells.

Altered vascular permeability and subsequent pulmonary oedema are perhaps some of the most significant characteristics of ALI patients^21^,^22^. The foreign pathogen-induced excessive activation of inflammatory mediators, such as LPS or TNF-α, is a known key factor that triggers endothelial cell activation, MT disassembly, and barrier dysfunction, and these changes are firmly demonstrated in our study (Fig. 1 and Fig. 2). These effects are implicated in the pathogenesis of pulmonary oedema associated with ALI^10^,^23^. Accumulating data indicate that LPS or TNF-α can induce MT disassembly and subsequently trigger microfilament rearrangements in endothelial cells; these rearrangements can be either dependent on or independent of MLCK and Rho, and the changes lead to cell contraction, cell shape changes, intercellular gaps, and microfilament stress fibre formation^9^,^10^. However, the manner in which LPS or TNF-α induces MT disassembly and regulates endothelial barrier function is still not well elucidated.

Members of the MAPs family include the neuronal proteins MAP2 and tau, which are primarily localized to the axon and the dendrites, respectively, and MAP4, the cytosolic MT-binding protein, which is present in all non-neuronal vertebrate cells^14^, is involved in a wide range of physiological and cellular functions (e.g., mitosis, myogenesis, and locomotion, the developmental regulation of the heart, and the maintenance of cytoskeletal stability)^19^,^24^,^25^. Once phosphorylated, MAP4 can dissociate from tubulin, leading to MT instability^12^,^13^. However, whether MAP4 plays an important regulatory role in HPMECs MT dynamics, which in turn affect endothelial barrier function, and the exact underlying mechanism remain unclear. In our study, we first demonstrated that LPS or TNF-α can induce robust phosphorylation of MAP4 at S696 and S787 in a time-dependent manner (Fig. 3a); these sites were recognised as the critical phosphorylation sites in the MT-binding domain^11^,^19^,^20^. Subsequently, the involvement of MAP4 phosphorylation in endothelial barrier regulation was directly demonstrated by the fact that the MAP4 (Ala) mutant, a constitutively dephosphorylated form of MAP4, can inhibit the LPS- or TNF-α-induced hyperpermeability, MT network remodelling, and MT disassembly (Fig. 3c, d, e and f). These results confirm that MAP4 phosphorylation regulates MT dynamics and vascular permeability in inflammation. Although, there is evidence to show the critical role of MAP4 in regulating vascular permeability in vitro experiment, the evidence of the effect and regulated mechanism of MAP4 in vivo experiment should further confirmed in future. Additionally, it should be noted that MTs interact with another component of the cytoskeleton, microfilaments^26^,^27^; this interaction successively activates GEF-H1, Rho/ROCK, and MLC phosphorylation under stress^28^,^29^. Whether MAP4 regulates the endothelial barrier function through MTs or cross-talking between MTs and microfilaments requires further study.

An important question arises as to which signalling pathway in lung endothelial cells is responsible for the MAP4 phosphorylation-dependent MT disassembly under inflammatory conditions. Our previous studies have indicated that p38/MAPK is the upstream kinase involved in MAP4 phosphorylation, as well as the p38/MAPK involved in the regulation of endothelial barrier function^11^,^16^,^17^,^30^. In this study, we first focused on the role of the p38/MAPK signalling pathway and identified a robust activation of this pathway after LPS or TNF-α stimulation in a time-dependent manner (Fig. 4a). Then, we tested the possibility that p38/MAPK activation could also be responsible for the MAP4 phosphorylation-dependent MT disassembly and the hyperpermeability in HPMECs. As we expected, MKK6 (Glu), which exhibits persistent activation of p38/MAPK, induced the MAP4 phosphorylation of S696 and S787 and its dissociation with tubulin (Fig. 4b and 4c), and this phosphorylation was followed by MT depolymerisation and elevated vascular permeability (Fig. 5a, b, and c). In contrast, SB203580, the p38/MAPK inhibitor, abolished the MAP4 phosphorylation and its dissociation with tubulin (Fig. 4c), promoted MT remodelling, and reduced the hyperpermeability of HPMECs (Fig. 5a, b, and c). Collectively, these data indicate that p38/MAPK is both required and sufficient for mediating the LPS- or TNF-α-induced lung vascular permeability via MAP4 phosphorylation and MT disassembly.

It is well known that LPS or TNF-α may induce endothelial apoptosis through a caspase-dependent pathway; this apoptosis could also lead to hyperpermeability in endothelial cells^31^,^32^,^33^. Additionally, our laboratory confirmed that phosphorylated MAP4 translocates to mitochondria and mediates hypoxia-induced cardiac apoptosis^13^. In general, there may be cross-talk between the vascular hyperpermeability induced by MT disassembly and that induced by apoptosis^10^,^34^. To further investigate whether the hyperpermeability induced by MAP4 phosphorylation and MT depolymerisation develops independently from apoptosis, the HPMECs were pretreated with the irreversible and cell permeable inhibitor of caspase-3^35^ to block the LPS- or TNF-α-induced caspase-dependent apoptosis (Supplementary Fig. S2a). Remarkably, pretreatment with an inhibitor of caspase-3, MAP4 (Ala) overexpression, and SB203580 pretreatment showed a more protective effect against endothelial barrier permeability compared with that of the CMV-null under the LPS or TNF-α challenge (Supplementary Fig. S2b); this finding suggested that MAP4 phosphorylation increased the lung vascular permeability in a MT-dependent manner independent of apoptosis.

In summary, we conclude that p38/MAPK-induced MAP4 phosphorylation plays a critical role in the LPS- or TNF-α-induced MT disassembly and the subsequent vascular hyperpermeability. Our data suggest that MAP4 may be a useful target for the specific modulation of inflammation-induced hyperpermeability in ALI.

## Methods

### Cell culture and reagents

Human pulmonary microvascular endothelial cells (HPMECs, ScienCell Research Laboratories, San Diego, California, USA) were grown in 6-well culture plates and maintained for 72 hr in endothelial cell medium (ECM) with 5% foetal bovine serum (FBS, ScienCell Research Laboratories), 1% Endothelial Cell Growth Supplement (ECGS, ScienCell Research Laboratories), and 1% penicillin/streptomycin (P/S) solution (ScienCell Research Laboratories, 10000 U/ml penicillin and 10000 μg/ml streptomycin) before the LPS and TNF-α treatments. The p38/MAPK inhibitor SB203580 (5 μM, Calbiochem, Germany), taxol (1 μM, AppliChem, Germany), or an irreversible and cell permeable inhibitor of caspase-3 (10 μM, Z-DQMD-FMK, APExBIO, USA) was added to these cultures, which were incubated at 37°C for 1 hr before the LPS and TNF-α treatments under serum-free conditions. LPS (Sigma, USA), TNF-α (Human, Protein Specialist, USA), protein A/G-Sepharose (sc-2003, Santa Cruz), HA (Sigma-Aldrich, USA), rabbit polyclonal anti-MAP4 (1:1000, Bethyl Laboratories, USA), mouse monoclonal anti-porin (1:2000, Calbiochem, Germany), rabbit polyclonal α-tubulin (1:50, Proteintech, USA), α-tubulin (1:1000, Santa Cruz Biotechnology, USA), P38/MAPK (1:1000, Cell Signaling Technology, USA), p-P38/MAPK (1:1000, Thr180/182, Cell Signaling Technology, USA), GAPDH-HRP (1:5000, Proteintech; USA), and p-MAP4 (S768) (1:1000, Biolegend, USA) were used. We raised the rabbit polyclonal antibody against p-MAP4 (S696, 1:500) or p-MAP4 (S787, 1:500) using the C-terminal 14 amino acids (PNKEPPP(pS)PEKKAK or KVAEKRT(pS)PSKPSSA and the respective non-phosphorylated peptides) conjugated to bovine serum albumin (BSA). The preparation and verification of the antibodies are described in the supplementary information (Supplementary Fig. S1b).

### Site-directed mutagenesis of MAP4 and MKK6 (Glu) recombinant adenovirus construction and transduction

Primers were designed to generate the MAP4 point mutations S696G, S768G, and S787G or S696A, S768A, and S787A through PCR reactions using the QuikChange® Multi Site-Directed Mutagenesis Kit (200514, Stratagene) (Supplementary Table S1). To evaluate the effects of signalling proteins, we constructed a recombinant adenovirus that expressed the mutated, constitutively activated human mitogen-activated protein kinase 6 (MKK6). The overexpression of constitutively activated MKK6 can selectively and constitutively activate p38/MAPK^36^. The pcDNA3 MKK6 (Glu) plasmid was developed from the Addgene plasmid 13518 by R. Davis, University of Massachusetts, Worcester, MA^37^, and the recombinant adenoviruses were prepared using the AdMax™ system (Microbix, Ontario, Canada) according to the manufacturer's instructions. After transfection of the endothelial cells with adenoviruses for 72 hr, the cells were assessed by Western blot, dextran permeability, transendothelial electrical resistance (TER), and immunofluorescence measurements.

### Measurement of the permeability to FITC-dextran

Cells were grown on Transwell compartments. Transwell tracer experiments with fluorescent dextran^38^ were performed using the 24-well Transwell system (Greiner Bio-One, 0.4-μm pore size, 6.5-mm diameter, transparent, Costar). HPMECs (1 × 10^5^ cells/well) were plated in the Transwell chambers; the volumes of culture media in the upper and lower compartments were 100 and 600 μl; and the cells were grown for 4 days and replaced with serum-free medium for 1 hr before treatment. The permeability was measured by the addition of FITC-dextran (40 kDa; Sigma-Aldrich) for 1 hr.

### Measurement of transendothelial electrical resistance

The cellular barrier properties were assessed by measuring the TER across confluent HPMECs using Millicell-ERS (MERS00002, Millipore, USA)^38^. The 24-well Transwell system was inserted into the 24-well plate. HPMECs were plated in Transwell chambers; the volumes of culture medium in the upper and lower compartments were 100 and 600 μl, respectively, and the cells were grown for 4 days and replaced with serum-free medium for 1 hr before treatment. The electrode tips were inserted into the upper compartment and the lower compartment, respectively, and the resistance (R sample) was measured; the value for the blank well represented the blank resistance (R blank). The following calculation was performed: endothelial monolayer resistance = (R sample-R blank)*effective membrane area (the area of a 24-well Transwell chamber is 0.336 cm^2^).

### Immunofluorescence and image analysis

Cells were grown on glass coverslips and cultured as described above. The cells were then fixed in 4% paraformaldehyde for 20 min, permeabilised with 0.1% Triton X-100 in PBS for 25 min, and blocked in 10% goat serum for 1.5 hr. To obtain the MT structure, rabbit anti-α-tubulin primary antibodies (1:50, Proteintech, USA) were diluted with PBS, and the coverslips were incubated at 4°C overnight. The coverslips were washed in PBS and then incubated with goat anti-rabbit secondary antibodies conjugated to cyanine 3 (Cy3, 1:100) for 1 hr at 37°C. The cells were imaged using confocal microscopy (TCS-NT, Leica, Wetzlar, Germany).

### Extraction and quantification of tubulin fractions

The free and polymerised tubulin fractions were isolated using a method previously described by Putnam et al.^39^. Cells grown in 6-well culture plates were washed twice with a MT stabilisation buffer (MTSB, 37°C) containing 0.1 M piperazine-N,N′-bis (2-ethanesulfonic acid, pH 6.8) (PIPES), 2 mM ethylene glycol-bis (β-aminoethylether)-N,N,N′,N′-tetraacetic acid (EGTA), 2 mM ethylenediaminetetraacetic acid (EDTA), 0.5 mM MgCl_2_, and 20% glycerol. The cells were incubated with MTSB + 0.1% Triton X-100 + protease inhibitor cocktail (1:200, Sigma-Aldrich) + phenylmethylsulphonyl fluoride (PMSF, 1:200, Sigma-Aldrich) + phosphatase inhibitor cocktail (1:200, Sigma-Aldrich). The polymerised and free tubulin fractions were quantified using Western blot analysis.

### Western blot analysis

The free and polymerised tubulin fractions prepared as described in the above section were probed using mouse anti-α-tubulin (1:1000, Santa Cruz Biotechnology, USA) antibody; the GAPDH-HRP (1:5000, Proteintech, USA) in the supernatant was chosen as the internal control for the free tubulin, and the mouse anti-Porin 31HL (Ab-2) (1:2000, Calbiochem, Germany) in the Triton X-100 insoluble fraction was chosen as the control for polymerised tubulin. The tubulin fractions and whole cells were stimulated and then lysed. The protein extracts were separated using sodium dodecyl sulphate-polyacrylamide gel electrophoresis (SDS-PAGE), transferred to a polyvinylidene fluoride (PVDF) membrane, and probed with specific antibodies as follows: p38 (1:1000, Cell Signaling Technology, USA), p-P38 (1:1000, Cell Signaling Technology, USA), MAP4 (1:1000, Bethyl, USA), p-MAP4 (S768, 1:1000) (Biolegend, USA), and rabbit polyclonal antibody against p-MAP4 (S696, 1:500) or p-MAP4 (S787, 1:500). As a loading control, GAPDH was probed and visualised. The immunocomplexes were visualised and quantified using an enhanced chemiluminescence detection kit (Amersham Pharmacia, Piscataway, NJ) and horseradish peroxidase-conjugated secondary antibodies.

### Immunoprecipitation

To search for the protein interacting with MAP4 and tubulin, cells were lysed in RIPA buffer with a protease inhibitor tablets. α-tubulin (Santa Cruz Biotechnology) antibody was incubated with cell lysate for 6 h at 4°C, then the complexes precipitated with protein A/G-Sepharose (sc-2003, Santa Cruz) overnight at 4°C. The precipitates were washed 5 times with PBS at 0°C and separated by SDS/PAGE and probed by rabbit anti-MAP4 (A301-489A, Bethyl Laboratories) antibody using Western blotting.

### Apoptosis assay

An In Situ Cell Death Detection Kit (11684795910, Roche) was used for the apoptosis assay. Fifteen representative images of each group were randomly chosen using an Olympus microscope (1 × 71, Japan), and the mean fluorescence intensity was analysed using Image-Pro Plus 5.0 software. The experiments were repeated 3 times, and the mean values were calculated.

### Statistical analysis

Statistical analysis of the data was performed using SPSS (Statistical Package for the Social Sciences) 13.0 software, and the significance was evaluated by one-way analysis of variance (ANOVA) followed by post hoc tests. *P* values < 0.05 were considered statistically significant.

## Author Contributions

Y.H. and B.C. supervised the work; L.L., J.H. and Y.H. designed the experiments with help from D.Z.; L.L., J.H., Q.Z., T.H. and X.L. performed the experiments; L.L., X.Y. and H.M. analysed the data; and L.L., J.H., Y.H. and B.C. co-wrote the manuscript. All authors discussed the results and commented on the manuscript.

## Supplementary Material

Supplementary Information supplementary information
