# Supplementary material for: P38/MAPK contributes to endothelial barrier dysfunction via MAP4 phosphorylation-dependent microtubule disassembly in inflammation-induced acute lung injury
Source: Sci Rep. 2015 Mar 9;5:8895. doi: 10.1038/srep08895 (PMC4352893; doi:10.1038/srep08895)
Supplement: Supplementary Information [file srep08895-s1.doc]

**P38/MAPK contributes to endothelial barrier dysfunction via MAP4 phosphorylation-dependent microtubule disassembly in inflammation-induced acute lung injury**

Lingfei Li^1^, Jiongyu Hu^2^, Ting He^1^, Qiong Zhang^1^, Xu Yang^3^, Xiaodong Lan^1^, Dongxia Zhang^1^, Hao Mei^4^, Bing Chen^2^*, Yuesheng Huang^1^*

^1^ Institute of Burn Research, State Key Laboratory of Trauma, Burns and Combined Injury, Southwest Hospital, Third Military Medical University, Chongqing, China, ^2^ Endocrinology Department, Southwest Hospital, ^3^ Institute of Respiratory Diseases, Xinqiao Hospital, Third Military Medical University, Chongqing, China, ^4^ Department of Biostatistics in the School of Public Health, Yale University.

Lingfei Li and Jiongyu Hu contributed equally to this work.

*Corresponding authors: yshuang.tmmu@gmail.com or chenbing3@medmail.com.cn

**Supplementary Information**

**Supplementary Table**

**Table S1** Primers used to generate mutant MAP4.

| Mutant sites | Primer Sequence | |
| --- | --- | --- |
|  | Reverse | Forward |
| S696G | CTTTTCTGGTTCTGGTGGTAGCTCCTTGTTTG | CTACCACCAGAACCAGAAAAGAAAGCAAAG |
| S768G | GGTAGGGTCTCAGGCCTGGCAGTAGCAGTG | GGTAGGGTCTCAGGCCTGGCAGTAGCAGTG |
| S787G | TGGATGGCTCGGTCCGCTTTTCAGCAAC | AAGCGGACCGAGCCATCCAAGCCTTCATC |
| S696A | CTTTTCTGGAGCTGGTGGTAGCTCCTTG | CAAGGAGCTACCACCAGCTCCAGAAAAG |
| S768A  S787A | GGAGCGGTCCGCTTTTCAGCAACCTTAGTTTCTGTAATTGGCTTTGGCTTCAGGTCTCTTGCAGGTAGGGTAGCAGGC | GCCTGCTACCCTACCTGCAAGAGACCTGAAGCCAAAGCCAATTACAGAAACTAAGGTTGCTGAAAAGCGGACCGCTCC |

**Supplementary Figure Legends**

**
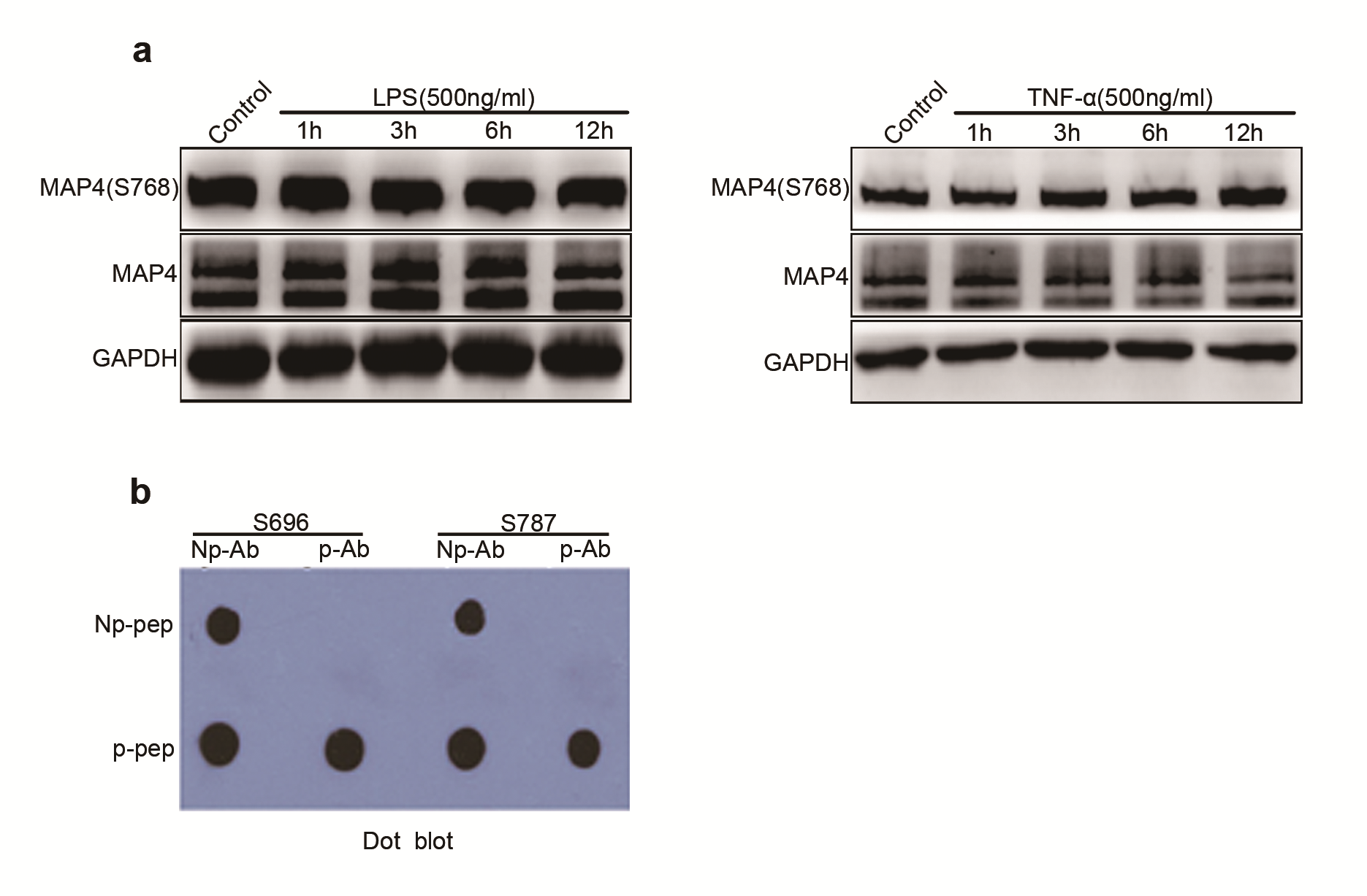
**

**Figure S1 LPS- or TNF-α-induced MAP4 phosphorylation in endothelial cells.** (a) LPS or TNF-α (500 ng/ml) was used to treat HPMECs, and Western blotting was used to assess the MAP4 (S768) and MAP4 levels (n=3). (b) Preparation and verification of anti-p-MAP4 (S696) and anti-p-MAP4 (S787) polyclonal antibodies. Np-pep: non-phospho-peptide, p-pep: phospho-peptide, Np-Ab: non-phospho-antibody, p-Ab: phospho-antibody.


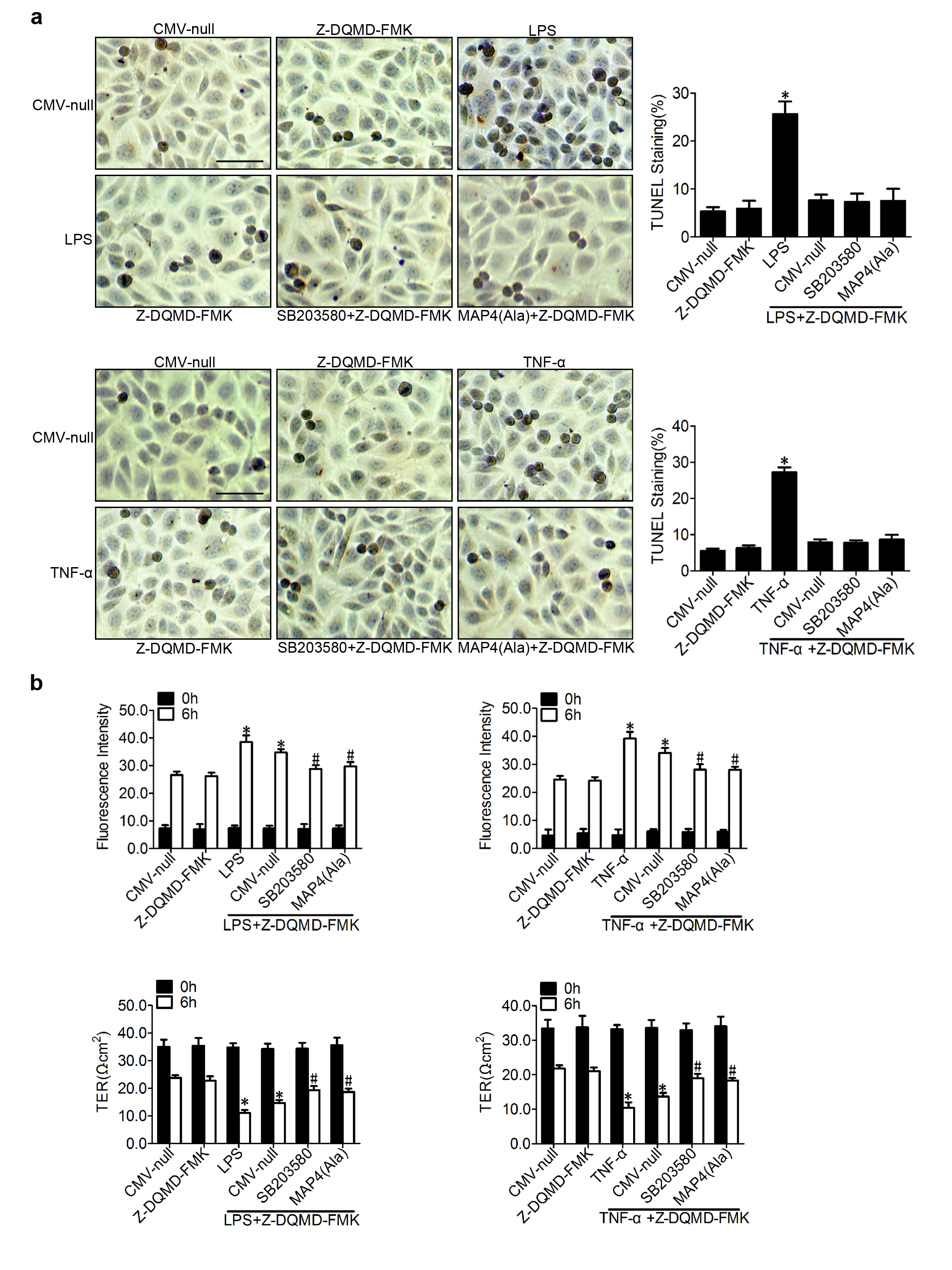


**Figure S2 LPS- or TNF-α-induced permeability develops independently of apoptosis.** (a) Representative images showing cell apoptosis using TUNEL (terminal deoxynucleotidyl transferase dUTP nick-end labeling) staining. The Z-DQMD-FMK, an inhibitor of caspase-3 (10 µM), SB203580 (5 µM), and MAP4 (Ala) overexpression were applied as indicated before LPS or TNF-α (500 ng/ml) treatment. Bar, 100 μm. The data are represented as the mean ± SEM (n=3). *P < 0.05 vs. CMV-null. (b) The Z-DQMD-FMK, an inhibitor of caspase-3 (10 µM), SB203580 (5 µM), and MAP4 (Ala) overexpression were applied as indicated before LPS or TNF-α (500 ng/ml) treatment. The permeability of endothelial cells was assessed by measuring the influx of FITC-conjugated dextran and the TER across the cells. The data are represented as the mean ± SEM (n=3). *P < 0.05 vs. CMV-null; **^＃^**P < 0.05 vs. the LPS/TNF-α and LPS/TNF-α+Z-DQMD-FMK groups.
